# Shifting the paradigm of type 1 diabetes: a narrative review of disease-modifying therapies

**DOI:** 10.3389/fendo.2024.1477101

**Published:** 2024-11-06

**Authors:** Alexander J. O’Donovan, Seth Gorelik, Laura M. Nally

**Affiliations:** ^1^ Yale University School of Medicine, Department of Pediatrics, New Haven, CT, United States; ^2^ Bowdoin College, Brunswick, ME, United States

**Keywords:** type 1 diabetes, stage 1 type 1 diabetes, stage 2 type 1 diabetes, stage 3 type 1 diabetes, teplizumab, disease-modifying therapies

## Abstract

A new diagnosis of type 1 diabetes (T1D) may be accompanied by numerous lifelong financial, emotional, and physical challenges, thus advancements in therapies that can delay the onset of clinical disease are crucial. T1D is an autoimmune condition involving destruction of pancreatic beta cells leading to insulin deficiency, hyperglycemia, and long-term insulin dependence. The pathogenesis of T1D is classified into stages, with the first signal being the detection of autoantibodies without any glycemic changes. In the second stage, dysglycemia develops without symptoms, and in stage 3, symptoms of hyperglycemia become apparent, and at this time a clinical diagnosis of T1D is made. As a greater understanding of these stages of T1D have evolved, research efforts have been devoted to delaying the onset of clinical disease. To date, only one medication, teplizumab, has been approved by the Food and Drug Administration (FDA) for the treatment of stage 2 T1D. This narrative review present published trials and ongoing research on disease modifying therapies (DMT) in T1D, the mechanisms of action for each therapy, and the stages of T1D that these interventions are being studied.

## Introduction

Type 1 diabetes (T1D) is an autoimmune condition that results in the destruction of insulin producing beta cells in the pancreas by CD4^+^ and CD8^+^ T cells and macrophages infiltrating the islets of Langerhans (1). Children with T1D commonly present with symptoms of polyuria, polydipsia, and weight loss, with about one-third of children presenting with diabetic ketoacidosis (2). Diagnostic criteria for diabetes include a fasting blood glucose concentration greater than or equal to 126 mg/dL, a random blood glucose concentration greater than or equal to 200 mg/dL with symptoms of hyperglycemia, a 2-hour glucose level of >200 mg/dL during an oral glucose tolerance test, or a glycated hemoglobin (HbA1c) greater than 6.5%. The exact trigger for the development of T1D is not well understood, however, a growing consensus suggests a convergence of genetic predisposition and environmental triggers in its pathogenesis. T1D accounts for about 10% of all cases of diabetes worldwide, and it occurs most commonly in people of European descent (1). A study by Gregory et al. found that in 2021, there were about 8.4 million individuals worldwide with T1D, and that by 2040, this number was expected to reach 13.5-17.4 million (3). The predicted rapid rise in cases of T1D coincides with the belief that the environmental effect on susceptibility genes plays a role in its epidemiology (1).

### Autoantibodies and screening for T1D

Regardless of the extent environmental and genetic causes are instigating a higher prevalence of T1D worldwide, an autoimmune response eventually occurs. The characterization of this autoimmune response has been known since the identification of autoantibodies in patient serum binding to islet cells dating back to 1974 (4). Identification of islet cell antibodies (ICA) sparked new research using advanced techniques, such as molecular cloning, gel electrophoresis, polymerase chain reaction, and DNA microarray analysis to discover more than ten target antigens related to the immune reaction (5). In 1983, the insulin autoantibody (IAA) was discovered in patients with newly diagnosed T1D (6). Following this, three additional autoantibodies were discovered to aid in screening, analysis, and prediction of T1D: GAD autoantibodies (GADA), discovered in 1990 (7), tyrosine phosphatase-like protein IA-2 autoantibodies (IA-2A), discovered in 1994 (8), and zinc transporter 8 autoantibodies (ZnT8A), discovered in 2007 (9). The type, number, and timing of developing autoantibodies improve predictions about timing of the onset of clinical disease and how the combination of autoantibodies predicts who may or may not respond to preventative therapies (10).

Most screening programs to identify individuals at risk for T1D, such as TrialNet and INNODIA, target relatives of people already diagnosed with T1D in an effort to improve yield and feasibility of using these autoantibodies as the screening tool. This, however, contradicts the fact that over 90% of those who go onto develop T1D do not have a family history (11). These programs have started to include monitoring or screening at risk individuals in the general population, now opting for online consent and optional at-home test kits. In total, the number of individuals without a relative with T1D who have been screened is greater than the number of relatives (11).

### Stages of T1D

Multiple prospective, longitudinal studies have identified T1D pathogenesis as a continuum of disease that occurs sequentially at different rates through three separate stages prior to the onset of symptoms (12). While diabetes has historically been diagnosed secondary to symptoms associated with the onset of hyperglycemia, the screening of autoantibodies can now be used to predict risk of developing T1D. The presence of known T1D-associated antibodies and presence of dysglycemia can place screened individuals in one of the three stages. Stage 1 occurs with the presence of two or more T1D-associated autoantibodies with otherwise normal glucose levels. The transition from Stage 1 to Stage 2 occurs when they develop dysglycemia. Stage 2 T1D is notable for loss of beta cell function, leading to elevated fasting plasma glucose levels, impaired glucose tolerance, or mildly elevated HbA1c (12). Stage 3 involves developing clinical symptoms of T1D, including polyuria, polydipsia, or weight loss with hyperglycemia, but still have insulin secretion (12).

## Methodology

A comprehensive literature review was conducted within PubMed utilizing the search terms “type 1 diabetes” and “disease modifying therapies.” To identify specific medications currently under investigation, additional searches were conducted on ClinicalTrials.gov using the condition filter “type 1 diabetes” and the search terms “beta cell preservation” and “disease modifying.” Breakthrough T1D (formerly the Juvenile Diabetes Research Foundation) and TrialNet websites were also reviewed to explore discussions on upcoming clinical trials. Identified medications were then searched in PubMed for publications. Given the relative paucity of literature in this field, exclusion criteria were fairly limited. However, a preference was given to medications demonstrating successful treatment outcomes. There were no limitations based on region of study or population studied. Information for the background studies was located through PubMed by employing the search terms “staging AND type 1 diabetes,” and “antibodies AND type 1 diabetes.” In total, 14 studies were included (**Table 1**).

**Table 1:** Published clinical trials of disease modifying therapies in type 1 diabetes.

Medication Class	Studied Medications	Stage of T1D Studied	Longest Follow-Up	Results of Clinical Trial*
**Anti-CD3 Monoclonal Antibodies**	*Teplizumab*	2	2 years	At 2 years, patients in the treatment group had less reduction in MMTT-stimulated C-peptide AUC when compared to the control group (17).
		3	2.5 years	At a median follow up of 2.5 years, 50% of the teplizumab-treated population remained in stage 2 T1D compared to 22% of the placebo group (21).
**Anti-CD20 Monoclonal Antibodies**	*Rituximab*	3	1 year	At 1 year, the mean MMTT-stimulated C-peptide AUC was significantly higher in the rituximab group than in the placebo group (23).
**Anti-IL-21 & GLP-1 agonists**	*Anti-IL-21 & Liraglutide*	3	54 weeks	At 54 weeks, those receiving Anti-IL-21 and liraglutide had 48% higher MMTT-stimulated C-peptide levels when compared to placebo, representing only a 10% decrease from baseline (72)
**Anti IL-12 & IL-23 Monoclonal Antibody**	*Ustekinumab*	3	1 year	At 1 year, those receiving the intervention had 49% higher MMTT-stimulated C-peptide levels (27)
**Dimeric Fusion Protein**	*Alefacept*	3	2 years	At 2 years, the alefacept group had lower insulin requirements, fewer hypoglycemic episodes and higher MMTT-stimulated C-peptide levels when compared to the control group (30)
**Anti-Thymocyte Globulins (ATG)**	*Thymoglobulin*	3	2 years	A 2-year MMTT-stimulated C-peptide AUC was significantly elevated in ATG versus placebo, but not in ATG+GCSF versus placebo. Both ATG and ATG+GCSF were associated with reduced HbA1c at 2 years (32).
**Calcium Channel Blockers**	*Verapamil*	3	1 year	The treatment group had a 30% higher MMTT-stimulated C-peptide AUC (45).
**CTLA-4 Analogs**	*Abatacept*	3	2 years	At the 2 year follow up, MMTT-stimulated C-peptide AUC was found to be 59% higher in the treatment vs placebo group (40).
**JAK Inhibitors**	*Baricitinib*	3	48 weeks	Daily treatment over 48 weeks was associated with an increased meal-stimulated mean C-peptide level (36).
**Tumor Necrosis Factor Alpha (TNF-α) Blockers**	*Golimumab*	3	1 year	At 1 year, the MMTT-stimulated C-peptide AUC remained higher in the treatment versus placebo group (37). C-peptide AUC decreased 12% with golimumab compared to 56% with placebo.
**Tyrosine Kinase Inhibitors**	*Imatinib*	3	2 years	The treatment group had a higher MMTT-stimulated C-peptide AUC at 1 year, but this effect was not sustained at 2 years (41).
**Neurotransmitter and antigen-based therapy**	*GABA and GAD-alum*	3	1 year	No change in glycemia, fasting or meal-stimulated C-peptide AUC at 1 year. Mean fasting glucagon levels did not increase in the GABA or GABA/GAD-alum groups and meal-stimulated glucagon levels were lower in the intervention groups (50)
**Autologous Dendritic Cell Therapy**	*AVT001*	3	1 year	At 1 year, there were no differences in HbA1c or insulin dose, but there was less decline in C-peptide production (52)
**Autologous Mesenchymal Stem Cells (MSC)**	*Autologous bone marrow derived MSCs*	3	1 year	Those receiving MSCs had reductions in level 1 and level 2 hypoglycemia, and fewer hypoglycemia events. Earlier treatment (within the first year) was associated with lower HbA1c levels at 1 year when compared to later treatment (at least 1 year after T1D diagnosis) (53)

*AUC, Area Under the Curve; MMTT, mixed meal tolerance test; HbA1c, hemoglobin A1c; GCSF, granulocyte colony-stimulating factor.

To complement the initial literature search conducted within PubMed, a comprehensive exploration of ongoing and future clinical trials for disease-modifying therapies in early-stage (**Table 2**) and recent-onset (**Table 3**) T1D was undertaken. ClinicalTrials.gov was utilized as the primary platform for this investigation. The search strategy employed two filters: “condition” set to “diabetes mellitus, type 1” OR “type 1 diabetes” and a combination of search terms including “stage 1,” “stage 2,” “stage 3,” “disease modifying,” and “recent onset.” Exclusion criteria were applied to filter out withdrawn or terminated studies. Conversely, inclusion encompassed any study matching the aforementioned search terms with a trial status listed as “recruiting,” “active, not recruiting,” or “completed” but lacking posted results. In sum, 16 studies relevant to early-stage and recent-onset T1D, summarized in **Tables 2**, **3**, were identified through this search strategy. This approach aimed to provide a comprehensive overview of the current and emerging clinical trial landscape for T1D disease-modifying therapies.

**Table 2:** Ongoing, future, and completed clinical trials investigating disease modifying therapies in stages 1 and 2 type 1 diabetes.

Medication Class	Studied Medications	Stage of T1D Studied	Trial Details*
**Oral Insulin**	*Recombinant Human Insulin (rH-insulin crystals)*	1	Randomized, triple-blind, phase 2 trial (*NCT02620072*) evaluating oral insulin to prevent T1D progression in stage 1, high-risk children aged 2-12 years. The study will assess prevention of dysglycemia or diabetes, as measured by oral glucose tolerance test. Participants are followed for at least 24 months (55).
**Anti-CD3 Monoclonal Antibodies**	*Teplizumab*	2	A single-arm, open-label, multicenter, phase 4 trial (*NCT05757713*) evaluating the safety and pharmacokinetics of teplizumab in young children (aged 0-8 years) with stage 2 T1D. The study will also assess the development of anti-drug antibodies and neutralizing antibodies. Each participant’s involvement may extend up to approximately 26 months (56).
**Glucagon-like Peptide-1 (GLP-1) Receptor Agonists**	*GLP-1Ra with Teplizumab*	2	Early-phase 1, randomized, quadruple-masked, crossover trial (*NCT06338553*) assessing the safety and efficacy of a single GLP-1Ra dose in combination with teplizumab in participants with stage 2 T1D. Primary outcomes include changes in blood glucose levels, insulin function, and vascular health, as measured by multiple MMTTs conducted pre- and 3-5 months post-teplizumab treatment (57).
**Polyclonal antibody**	*Anti-Thymocyte Globulins (ATG)*	2	Phase 2, randomized, double-blind, placebo-controlled trial (*NCT04291703*) investigating low-dose ATG to prevent progression from stage 2 to stage 3 T1D in high-risk individuals. Participants are followed for up to 5 years (58).
**Antigen-specific Immuno-modulators**	*Diamyd*	1 & 2	Phase 2, randomized, parallel assignment, open-label trial (*NCT05683990*) evaluating Diamyd in children and adolescents aged 8-18 years with stage 1 or 2 T1D. Primary outcomes include safety and tolerability, assessed by hematology, clinical chemistry, metabolic status parameters (fasting C-peptide, HbA1c, fasting glucose) and urine analysis. Participants will be followed for 12 months (59).

*T1D, type 1 diabetes; MMTT, mixed meal tolerance test; HbA1c, hemoglobin A1c.

**Table 3:** Ongoing, future, and completed clinical trials investigating disease modifying therapies in recent-onset type 1 diabetes.

Class	Medication	Duration of T1D	Trial Details*
**Supercoiled plasmid vector**	NNC0361-0041	<48 months	Phase 1, placebo-controlled, double-blind, randomized, dose-escalation, sequential assignment trial (*NCT04279613*) evaluating the safety and tolerability of NNC0361-0041 plasmid in patients with T1D. Primary outcome measures include safety, as assessed by adverse event incidence, and efficacy, as determined by changes in C-peptide levels during MMTT from baseline to 12 months (60).
**Interleukin Inhibitors**	*Ustekinumab (Anti-Interleukin (IL)-12 and IL-23 Antibody)*	≤100 days	Phase 2/3, randomized, parallel assignment, double-blind, placebo-controlled trial (*NCT03941132*) assessing efficacy and safety of Ustekinumab in T1D. Primary outcome measures include baseline changes in 2-hour MMTT-stimulated C-peptide AUC, HbA1C, insulin use, and incidence of all adverse events. The follow up period is 12 and 18 months from the first dose (61).
**Interferons**	*Human Recombinant Interferon-Alpha (IFN-a)*	≤6 weeks	Phase 2, randomized, double-blind trial (*NCT00024518*) evaluating interferon-alpha in preserving residual endogenous insulin production. Primary outcomes include C-peptide levels and hemoglobin HbA1c. Participants are followed up at 3-month intervals over the course of 12 monthsr (62).
**Interleukin Agonists**	*Recombinant human IL-2*	≤ 3 months	Phase 2, randomized, quadruple-blind, parallel-assignment trial (*NCT02411253*) evaluating the efficacy and safety of low-dose IL-2 in preserving beta cell function in recent-onset T1D. Primary outcomes include change in C-peptide AUC, determined after a MMTT at month 12. The study duration is 24 months (63).
**Imotopes**	*IMCY-0098*	≤6 months	Phase 1, randomized, double-blind, sequential assignment trial (*NCT03272269*) assessing safety and immunogenicity of IMCY-0098 in participants with recent-onset T1D. Primary outcome measures include adverse events, changes in C-peptide production, HbA1c, and changes in IMCY-0098 specific T lymphocyte responses. Participants will be followed for 24 weeks post-enrollment (64).
**Stem Cell Therapy**	*Adipose Tissue-Derived Stem/Stromal Cells with* *Cholecalciferol*	≤4 months	Randomized, parallel assignment, open-label, prospective Phase 2 trial (*NCT03920397*) comparing adipose-derived stromal/stem cells plus cholecalciferol to cholecalciferol alone in patients with recent-onset T1D. Adverse effects will be recorded. In addition, glycated hemoglobin, insulin dose, frequency of hypoglycemia, glycemic variability, % of time in hyper and hypoglycemia and peak response of the C-peptide after the MMTT will be measured at three-month intervals for a 24-month period (65).
**Perinatal Tissue Derived Cells (PTDCs)**	*CELZ-201*	≤6 months	Phase 1/2a, randomized controlled trial (*NCT05626712*) evaluating CELZ-201 therapy in recent-onset T1D. Primary outcome measures include safety and efficacy, assessed by changes in C-peptide during a 4-hour MMTT, HbA1c, exogenous insulin requirements, and autoantibody levels. Study duration is 24 months (66).
**Vitamin D Analogs**	*Calcitriol*	≤3 months	Phase 2, randomized, double-blind trial (*NCT01120119*) evaluating calcitriol in preserving beta cell function in recent-onset T1D. Primary outcome measures include changes in fasting and stimulated C-peptide, insulin requirements and HbA1c. Participants are followed up for 24 months (67).
**JAK Inhibitors**	*Abrocitnib; Ritlecitinib*	≤100 days	Phase 2, multi-center, randomized, double-blind, parallel assignment, placebo-controlled trial (*NCT05743244*) comparing two JAK inhibitors in individuals with recent-onset T1D. The primary outcome of interest is the change in stimulated C-peptide production during the 12-month follow-up period (68).
**Polyclonal Regulatory T Cells**	*CD4^+^CD127lo/-CD25^+^ & Interleukin-2 (IL-2)*	>3 and <24 months	Phase 1, single-arm, open-label (*NCT02772679*) trial evaluating safety and preliminary efficacy of polyclonal regulatory T cells (Tregs) plus IL-2 in patients with T1D. Primary outcome measures include safety, changes in beta cell function (C-peptide in response to serial MMTT), glycemia (HbA1c), and Treg survival (69).
**Serine Protease Inhibitors (SERPINS)**	*Alpha-1 Antitrypsin; Glassia*	≤6 months	Phase 1/2, randomized, parallel assignment trial (*NCT01304537*) evaluating safety and efficacy of alpha-1 antitrypsin (AAT) in T1D. Primary outcomes include incidence of adverse events, beta cell function, exogenous insulin requirements, and HbA1c. Participants will be followed for 12 months post-enrollment (70).

*T1D, type 1 diabetes; AUC, area under the curve; MMTT, mixed meal tolerance test; HbA1c, hemoglobin A1c.

## Disease modifying therapies

**Figure 1:**
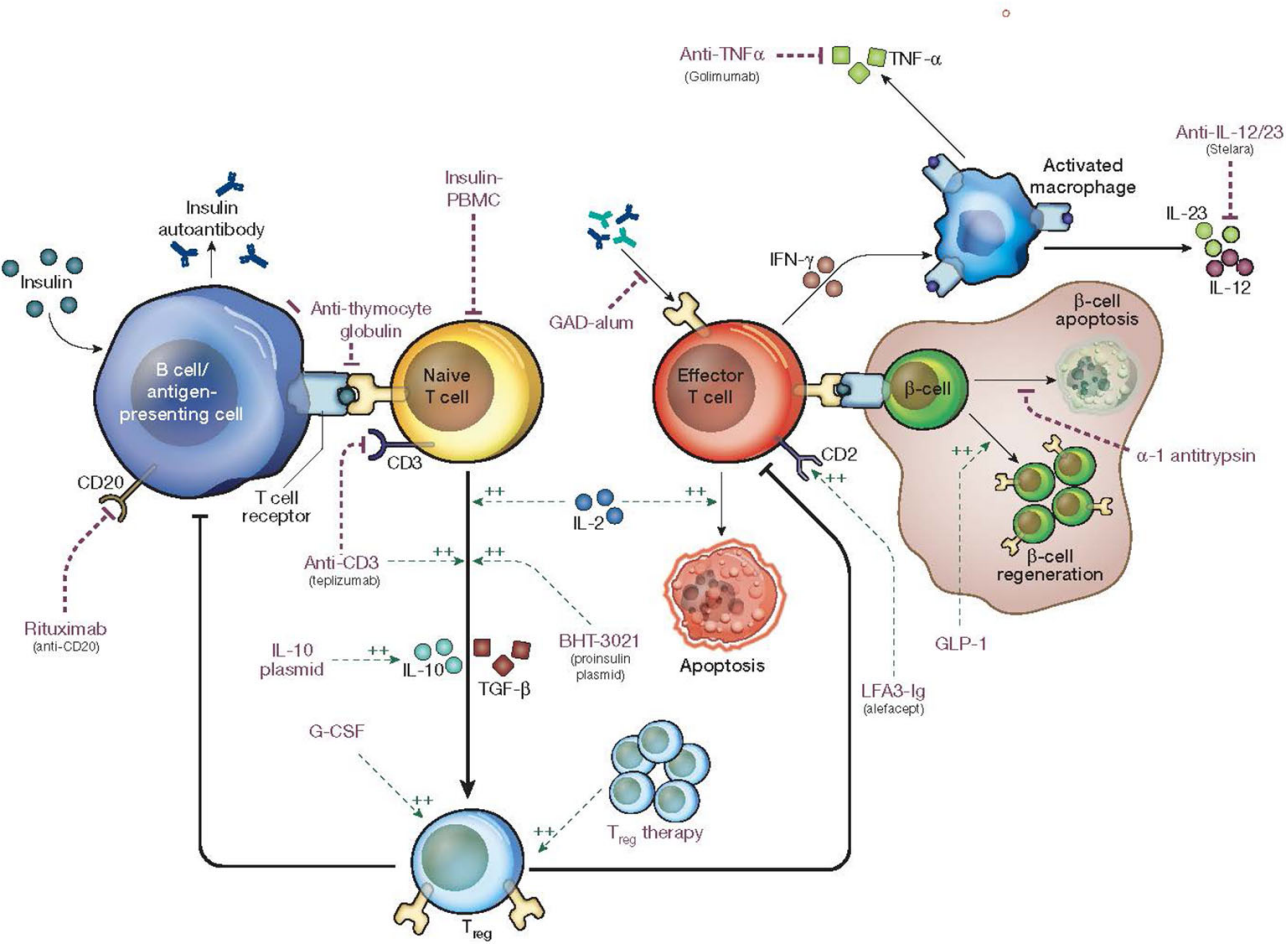
Mechanism of action of disease modifying therapies in type 1 diabetes. Edited with permission from Springer Nature (71).

With the classification of T1D into stages, therapies to intervene at each stage are becoming widely studied. Interventions that have the potential to preserve beta cell function may improve the metabolic and glycemic outcomes in new onset T1D (**Figure 1**). A majority of trials studying DMTs use C-peptide preservation to quantify responses (**Table 1**) (13).

### Anti-CD3 monoclonal antibodies (*Teplizumab*)

In November 2022, Teplizumab was the first drug approved by the FDA to delay the progression from stage 2 to stage 3 T1D. Teplizumab is a humanized anti-CD3 monoclonal antibody that can reduce T-cell activation, proliferation, and cytokine release *in vitro*. Early studies of the drug’s mechanism suggested that it could minimize the effects of CD8^+^ T cells involved in the autoimmune-related destruction of pancreatic beta cells (14). The initial studies using teplizumab were conducted in those with stage 3 T1D, where participants received either a randomized, placebo, or standard of care design (14).

The first phase 1/2 randomized controlled trial (*Study 1*) tested a single 14-day course of teplizumab in those with recently diagnosed T1D (15). Compared to standard of care, the teplizumab group had preserved beta cell function when comparing C-peptide levels during mixed-meal tolerance tests (MMTT). Following this initial study, the Autoimmunity-Blocking Antibody for Tolerance (AbATE trial), a randomized phase 2 trial (with a 14-day course of teplizumab administered 12 months after diagnosis of T1D also successfully reduced the decline in C-peptide response to a MMTT at 24 months from initial treatment when compared to the control group (15–18). Two phase 3 clinical trials for teplizumab (*Protégé* and *Encore*) tested three different dosing regimens of the drug over two courses that were 6 months apart with the end points of exogenous insulin use and HbA1c (19). However, the *Protégé* study was terminated for not meeting its primary endpoint. Finally, a fifth study (*Delay*) was a phase 2 trial that tested teplizumab in a cohort of patients recruited 4 to 12 months after T1D diagnosis who still had clinically significant levels of C-peptide (20). The onset of T1D was comparable to past studies that enrolled within 12 weeks of diagnosis. Endogenous insulin secretion was detectable in all interventions, consistent with preserved beta cell function (14).

Due to its success in stage 3 T1D, teplizumab was also studied in earlier stages of T1D. In *TN-10*, a randomized placed-controlled study including 76 participants with stage 2 disease and dysglycemia, suggested that teplizumab may delay beta cell degradation, although the change in magnitude was overall less than would be seen in Stage 3 T1D (20). A follow-up study completed at a median of 923 days after the initial study found that 50% of the teplizumab-treated population remained in Stage 2 T1D compared to only 22% of the control group. This change was attributed to partially exhausted memory T cells with reduced secretion in IFNγ and TNFα. This implies that a single course of teplizumab has a lasting affected in delaying stage 3 T1D in higher risk individuals (21). In a meta-analysis of 8 randomized, controlled trials including 866 patients with a clinical diagnosis of T1D who had received teplizumab, teplizumab use was found to be associated with decreased insulin use at 6, 12, and 18 months after diagnosis, and stimulated C-peptide AUC was higher at 12, 18, and 24 months (22). Thus, teplizumab has consistently showed improved endogenous insulin production when given during stage 2 or stage 3 diabetes.

### Anti-CD20 monoclonal antibodies (*Rituximab*)

B cells are involved in a wide array of T lymphocyte diseases and play an important role as antigen presenting cells, expressing high levels of MHC class II which influence escape of auto-reactive T-cells thought to trigger autoimmune conditions (23). CD20 is a protein expressed on B cells and is involved in the proliferation and differentiation of B cells into plasma cells. A TrialNet study (*TN05*) researched the effects of rituximab, a monoclonal antibody against CD20 that has been used in both oncologic and rheumatologic presentations in the past. This study was a randomized, double-blind study in participants between ages 8 and 40 with stage 3 T1D who had at least one type of detectable diabetes autoantibody. At 12 months, the mean C-peptide area under the curve (AUC) was significantly higher in the rituximab group than in the placebo group. The rate of decline of C-peptide levels was also significantly slower in the treatment group (23).

### Anti-IL-21 and GLP-1 Agonists (*Liraglutide*)

Interleukin-21 (IL-21), a cytokine produced by T cells, plays an important role in the trafficking and activation of autoreactive CD8+ T cells in the beta cell (72, 73), thus making it a potential therapy target in the prevention of T1D. In this study, Anti-IL-21, considered a milder, well-tolerated immunomodulatory agent, was tested alone and in combination with a GLP-1 agonist, liraglutide, which has been associated with decreased beta cell stress and preservation of insulin secretion. To test the isolated and synergistic effects on beta cell preservation, a randomized 4-arm placebo-controlled, doubledummy, double-blind phase 2 clinical trial evaluated the impact of IL-21 and liraglutide on C-peptide secretion over 54 weeks. Adults with T1D diagnosed within 20 weeks with at least two known T1D autoantibodies and residual beta cell function were included. Participants were randomly assigned equally to liraglutide, anti-IL-21, both, or placebo, receiving treatment over 54 weeks and monitored for another 26 weeks after the cessation of treatment. During the treatment period, C-peptide secretion decreased by 10% in the group receiving anti-IL-21 and liraglutide, compared to a 39% decrease with placebo. Further, Cpeptide secretion was 48% higher in the combination group when compared to the placebo group. No difference in C-peptide secretion was found when comparing single therapy with liraglutide or anti-IL-21 to placebo. During the 26-week observation period after cessation of therapy, no significant differences in C-peptide secretion, HbA1c, or total daily insulin dose were noted (72).

### Ustekinumab (*Stelara*)

Ustekinumab (Stelara), most commonly used in plaque psoriasis, psoriatic arthritis, and inflammatory bowel disease (24), is a monoclonal antibody that binds to the p40 receptor and inhibits IL-12 and IL-23 cytokines, preventing the differentiation of CD4^+^ cells into Th1 cells that produce IFN-gamma and Th17 cells that produce IL-17 (25). In a phase 1b open-label dose-finding study, it was found to reduce the percentage of circulating Th17, Th1, and Th17.1 cells and proinsulin-specific T cells that secreted IFN-γ and IL-17A and be safe for use in adults with T1D (26). Following this finding, a randomized, placebo-controlled, double-blinded, multi-center phase 2 study of ustekinumab (USTEKID Study) was conducted in adolescents who were diagnosed with T1D within 100 days and had at least 1 T1D autoantibody. Participants received 6 doses of ustekinumab over 48 weeks and were followed for 78 weeks following the first dose (27). At 12 months, those receiving the intervention had 49% higher meal-stimulated C-peptide levels and was also associated with lower levels of Th17.1 cells producing IL-17A, IFN-gamma, as well as B-cell stimulated Th17.1 cells (27).

### Alefacept

In order to closely target effector T cells involved in autoimmune beta cell destruction, investigators studied Alefacept, a fusion protein on IgG1 that binds to CD2 on CD4^+^ and CD8^+^ effector T cells (28). Alefacept targets memory-effector T cells, preventing T cell activation and proliferation while also inducing T cell apoptosis in select cells (29). The T1Dal study, a multicenter, randomized, double blind placebo-controlled trial, was conducted to compare two 12-week courses of alefacept with placebo in 49 individuals with recently diagnosed T1D (30). At 24 months, the group receiving alefacept had lower insulin requirements and 50% fewer episodes of hypoglycemia, however no meaningful differences in glycemia emerged. Not surprisingly, endogenous insulin secretion, measured by meal-stimulated 2- and 4-hour C-peptide AUC, was higher in the alefacept group when compared to the control group (30).

### Anti-thymocyte globulins (*Thymoglobulin*)

Anti-Thymocyte Globulins (ATG) have historically been used in the cases of bone marrow transplant, solid organ transplant, and aplastic anemia for over four decades, and these cases are associated with a nearly complete immune suppression. Initial studies of high dose ATG in T1D were unsuccessful in demonstrating clinical significance, which may be related to the dose-dependent depletion of CD4^+^ effector and regulatory cells (31). Later studies were completed using a low dose of ATG (Thymoglobulin) and ATG plus granulocyte colony-stimulating factor (GCSF). Following this study, Haller et al. tested low dose ATG in adolescents and young adults ages 12-45 with at least 1 autoantibody and were within 100 days of T1D diagnosis. They found that the 24-month MMTT stimulated C-peptide AUC was significantly higher in ATG versus placebo, but not in ATG+GCSF versus placebo. Both ATG and ATG+GCSF were associated with reduced HbA1c at 24 months (32).

Initial studies in non-obese diabetic (NOD) mouse models found that ATG plus GCSF demonstrated synergy and significant reversal of diabetes, likely due to the idea that ATG depletes pathogenic T cells while GCSF promotes regulatory T cells (32). The success of low-dose ATG is at least partially attributed to the fact that it was able to avoid long-term immunosuppression and maintain the beneficial regulatory functions of components like regulatory CD4^+^ T cells that are essential to immune tolerance. Low-dose ATG led to a decrease in the number of CD4^+^ T effector cells, an increase in the number in memory CD4^+^ T cells, and overall preservation of the more naïve CD8^+^ T cells (31).

### JAK inhibitors (*Baricitinib*)

The hyperexpression of HLA-I molecules on pancreatic beta cells has been accepted as one of the leading components in the pathogenesis of T1D. This increased expression draws the attention of autoreactive CD8^+^ T cells, which can accelerate autoimmune destruction. Interferons released by residual beta cells and autoreactive immune cells activate the JAK/STAT pathway, leading to more expression of genes involved in the autoimmune pathway (33).

In animal models, cytotoxic T cells that were deficient of *Tyk2*, a member of the JAK-STAT family, displayed overall reduced cytotoxicity. Treatment with a selective *Tyk2* inhibitor was also found to inhibit the expansion of autoreactive cytotoxic T cells, inflammation of beta cells, and onset of autoimmune T1D in NOD mice (34). Baricitinib, a JAK Inhibitor (JAKi) used in the treatment of rheumatoid arthritis (35), is one of the JAK inhibitors being studied in T1D. A phase 2, double-blind, randomized, placebo-controlled trial from Waibel et al. in 2023 found that daily treatment with baricitinib in patients within 100 days of diagnosis with stage 3 T1D over 48 weeks had a statistically significant change in mixed-meal stimulated mean C-peptide level, thus preserving beta cell function (36).

### Tumor necrosis factor alpha blockers (*Golimumab*)

The *TIGER* study was a randomized, double masked, multicenter interventional phase 2 clinical trial assessing the effects of golimumab in new onset T1D. Golimumab, a Tumor Necrosis Factor Alpha (TNF-α) blocker, or placebo was administered in participants within 100 days of diagnosis of stage 3 T1D and who had at least one diabetes-related autoantibody. At the end of 12 months, the C-peptide AUC remained significantly greater in the treatment versus control group. The mean percent decrease in mean 4-hour C-peptide AUC was 12% in the golimumab group and 56% in the placebo group, and this difference in C-peptide secretion was found as early as week 12 (37). There was no statistically significant difference between HbA1c and hypoglycemia between the two groups (37). A 24-month follow up study also found that there were trends in decreased insulin use, higher meal-stimulated peak C-peptide levels, and an increase in those in partial remission (insulin dose–adjusted HbA1C ≤ 9) in the golimumab treatment group (38).

### CTLA-4 analogs (*Abatacept*)

In order for a T-cell dependent B-cell response to occur, both a primary and secondary signal must be achieved. The first signal consists of a T-cell receptor binding to antigens presented by MHC class II molecules. A secondary signal consists of interactions between receptor-ligand pairs on T cells and antigen presenting cells that are non-antigen specific. The CD28/CTLA-4:CD80/CD86 costimulatory pathway is one of these pairs. CD28 and CTLA-4 are present on T cells while CD80 and CD86 are present on B cells. When CTLA-4 binds to CD80 and CD86, T-cell activation and proliferation is inhibited (39).

As CTLA-4 is a negative modulator for T-cell immunity, it serves as a method in which medication can become utilized. Abatacept, a CTLA-4 Analog, has been used successfully in conditions like psoriasis and rheumatoid arthritis. With the success abatacept has had in other presentations, it served as a good candidate for use in T1D as well. TrialNet completed a multicenter, randomized, double-blind, placebo-controlled trial (*TN09*) with the primary outcome of mean AUC serum C-peptide at a 24-month follow-up. Patients were required to have stage 3 T1D less than 100 days and have at least one diabetes-related autoantibody. At the 24-month follow up, C-peptide AUC was found to be 59% higher in the treatment vs placebo group, showing slowed reduction in beta cell function (40).

### Tyrosine kinase inhibitors (*Imatinib*)

Imatinib, a tyrosine kinase inhibitor most commonly used to treat chronic myeloid leukemia, was also examined in a multicenter, randomized, double-blind, placebo-controlled, phase 2 trial in participants within 100 days of diagnosis with stage 3 T1D, aged 18-45 years old, with at least one positive diabetes related autoantibody. Participants were given either imatinib or placebo daily for 26 weeks. The study achieved its primary endpoint, with a higher C-peptide AUC at 12 months in the treatment group versus placebo, however this effect was unfortunately not sustained at 24 months (41).

### Calcium channel blockers (*Verapamil*)

Verapamil, an antihypertensive calcium channel blocker, demonstrated the survival of insulin-producing beta cells and reversal of diabetes in mouse models (42). As diabetes develops, beta-cell TXNIP becomes overexpressed, triggering apoptosis of the beta cell (43). In murine models, verapamil reduced TXNIP expression and beta cell death and improved endogenous insulin production (43). To test the effect in humans, a randomized double-blind placebo-controlled phase 2 clinical trial in adults with a diagnosis of T1D within 3 months were given verapamil or placebo for 12 months. Both groups had similar HbA1c levels at the end of 12 months but those receiving verapamil had higher c-peptide production in response to MMTT at 3 and 12 months (44). Following this study, a double-blind, randomized clinical trial including 88 children and adolescents aged 7 to 17 years with newly diagnosed T1D was completed in 2023 (CLVeR Trial). Participants were treated within 31 days of diagnosis of stage 3 T1D and were randomized to daily verapamil or placebo for 52 weeks. Those receiving Verapamil had a 30% higher C-peptide AUC in response to a MMTT (45), consistent with increased endogenous insulin production. Thus, verapamil use was associated with preserved beta cell function in both pediatric and adults with a recent diagnosis of T1D.

### Gamma aminobutyric acid and glutamic acid decarboxylase

Gamma aminobutyric acid (GABA) is a neurotransmitter that serves an autocrine and paracrine role in islet cells, with *in vitro* studies in human islets suggesting that GABA increases insulin secretion from beta cells and may also have a regulatory role for alpha and delta cells (46). Likewise, some studies have suggested that glutamic acid decarboxylase (GAD-alum) therapy may slow the loss of insulin secretion in stage 3 T1D (47, 48). Combination therapy with GABA and GAD-alum has prolonged the lifespan in transplanted islet cells non-obese diabetic mice, signifying its potential as a therapeutic agent to prolong islet cell function in early T1D (49). In a randomized double blind randomized (2:1) trial, participants received either GABA alone, a combination of GABA and GAD, or placebo for 5 weeks. While there was no change in fasting or meal-stimulated C-peptide AUC at 12 months and no change in glycemia, mean fasting glucagon levels had increased by 16.8% in the control group and 0-0.4% in the intervention groups. Further, meal-stimulated glucagon levels were lower in the intervention groups (50). Thus, additional studies are needed to evaluate how these agents influence insulin and glucagon secretion.

### Novel autologous dendritic cell therapy

Regulatory T cells (Treg) are integral for maintaining immune tolerance, and abnormalities in CD8^+^ Treg pathway have been identified in those with T1D (51). In a combined phase 1/2 trial, a vaccine (AVT001) comprised of immature autologous dendritic cells that had been primed with an oligopeptide was designed to correct the defective CD8^+^ Treg pathway (52). The phase 1 portion of the randomized, double-blinded placebo-controlled study, the vaccine was administered to youth at least 16 years old within 12 months of T1D diagnosis and there were no serious adverse events during the 360 days of follow up. In the phase 2 study, there were no differences in HbA1c or insulin dose, but there was less decline in C-peptide production during the 360 day follow up, though the difference was small (52).

### Autologous mesenchymal stem cell transplantation

Autologous mesenchymal stem cells (MSCs) pose great promise as a therapeutic immunomodulatory and regenerative agent in the pathogenesis of T1D (53). MSCs are multipotent progenitor stem cells that have beneficial healing and anti-inflammatory properties without activating immune responses (54). In a triple-blinded parallel randomized placebo-controlled trial, children and young adults ages 8-14 with a diagnosis of T1D within the previous 6 weeks were randomized to receive 2 doses of autologous MSCs or placebo (0 and 3 weeks) (53). Safety criteria were met in the phase 1 portion for the study. There was a meaningful reduction in level 1 and level 2 hypoglycemia as well as fewer total hypoglycemia events in the MSC group. The intervention group also produced higher levels of anti-inflammatory cytokines that persisted over the 12-month study period and lower levels of the pro-inflammatory TNF-alpha (53). Likewise, earlier treatment (within the 12 months) when compared to later treatment (at least 12 months after T1D diagnosis) was shown to have a more pronounce impact on lower of A1c levels for 12 months (53).

## Ongoing/Future studies

As of August 2024, there are some additional studies about investigating DMT that could be used in recently diagnosed T1D (**Tables 2**, **3**). TrialNet is also conducting the TOPPLE T1D study, a placebo-controlled, double-blinded within cohorts, randomized, multiple ascending dose trial in assessing 12 weeks of once weekly dosing of the NNC0361-0041 plasmid, assessing C-peptide responses to multiple mixed-meal tolerance tests over 12 months. The intervention will be a recombinant supercoiled plasmid that encodes for four proteins: pre-proinsulin, transforming growth factor β1, IL-10, and IL-2 (**Table 3**).

## Discussion

The emergence of a diverse array of disease-modifying therapies, particularly within the biologics and immunotherapeutic domains, presents a promising landscape for T1D management. As detailed in **Tables 2**, **3**, a growing number of clinical trials are investigating interventions across various stages of T1D. While this review highlights a slight preponderance of studies focused on recent-onset T1D, the importance of early intervention cannot be overstated. Delaying the onset of T1D at earlier stages is associated with substantial benefits, especially for children, who may lose over 14 years of life expectancy if diagnosed before the age of 10 (20). Collectively, these emerging therapies can significantly improve health outcomes by addressing T1D across its entire disease trajectory.

The incidence of T1D continues to increase and rapid advancements are being made with preventative efforts to delay the onset of T1D. While only one medication, teplizumab, has been approved by the FDA in earlier stages of T1D, there are many other areas in the immune response in T1D that are being studied. Targeted therapies aimed at delaying the onset of T1D and preserving endogenous insulin secretion are vital to reducing the risk of severe long-term complications and have the potential to dramatically improve quality of life.
