# Corrigendum: Shifting the paradigm of type 1 diabetes: a narrative review of disease-modifying therapies

**DOI:** 10.3389/fendo.2024.1548761

**Published:** 2025-01-28

**Authors:** Alexander J. O’Donovan, Seth Gorelik, Laura M. Nally

**Affiliations:** ^1^ Yale University School of Medicine, Department of Pediatrics, New Haven, CT, United States; ^2^ Bowdoin College, Brunswick, ME, United States

**Keywords:** type 1 diabetes, stage 1 type 1 diabetes, stage 2 type 1 diabetes, stage 3 type 1 diabetes, teplizumab, disease-modifying therapies

In the published article, there was an error in the article title. Instead of “Shifting the paradigm of type 1 diabetes: a narrative review of disease modifying therapies”, it should be “Shifting the paradigm of type 1 diabetes: a narrative review of disease-modifying therapies”.

The authors apologize for this error and state that this does not change the scientific conclusions of the article in any way. The original article has been updated.

In the published article von Herrath M, Bain SC, Bode B, Clausen JO, Coppieters K, Gaysina L, et al. Anti-interleukin-21 antibody and liraglutide for the preservation of β-cell function in adults with recent-onset type 1 diabetes: a randomised, double-blind, placebo-controlled, phase 2 trial. *Lancet Diabetes Endocrinol*. 2021;9(4):212-224. doi:10.1016/S2213-8587(21)00019-X was not cited in the article.

In the published article McGuire HM, Walters S, Vogelzang A, Lee CMY, Webster KE, Sprent J, et al. Interleukin-21 is critically required in autoimmune and allogeneic responses to islet tissue in murine models. *Diabetes* (2011) 60:867–75. doi: 10.2337/db10-1157 was not cited in the article.

The citation has now been inserted in **Disease Modifying Therapies**, Anti-IL-21 Monoclonal Antibodies & GLP-1 Receptor Agonists (*Liraglutide*), Paragraph 1 and should read:

“Anti-IL-21 and GLP-1 Agonists (*Liraglutide*)

Interleukin-21 (IL-21), a cytokine produced by T cells, plays an important role in the trafficking and activation of autoreactive CD8+ T cells in the beta cell (72, 73), thus making it a potential therapy target in the prevention of T1D. In this study, Anti-IL-21, considered a milder, well-tolerated immunomodulatory agent, was tested alone and in combination with a GLP-1 agonist, liraglutide, which has been associated with decreased beta cell stress and preservation of insulin secretion. To test the isolated and synergistic effects on beta cell preservation, a randomized 4-arm placebo-controlled, double-dummy, double-blind phase 2 clinical trial evaluated the impact of IL-21 and liraglutide on C-peptide secretion over 54 weeks. Adults with T1D diagnosed within 20 weeks with at least two known T1D autoantibodies and residual beta cell function were included. Participants were randomly assigned equally to liraglutide, anti-IL-21, both, or placebo, receiving treatment over 54 weeks and monitored for another 26 weeks after the cessation of treatment. During the treatment period, C-peptide secretion decreased by 10% in the group receiving anti-IL-21 and liraglutide, compared to a 39% decrease with placebo. Further, C-peptide secretion was 48% higher in the combination group when compared to the placebo group. No difference in C-peptide secretion was found when comparing single therapy with liraglutide or anti-IL-21 to placebo. During the 26-week observation period after cessation of therapy, no significant differences in C-peptide secretion, HbA1c, or total daily insulin dose were noted (72).”

In the published article, there was an error in **Table 1** as published. We neglected to include a relevant study to the table, which also includes the newly added citation by von Herrath above. The corrected **Table 1** and its caption appear below.

**Table 1:** Published clinical trials of disease modifying therapies in type 1 diabetes.

*Medication Class*	*Studied Medications*	*Stage of T1D Studied*	*Longest Follow-Up*	Results of Clinical Trial*
**Anti-CD3 Monoclonal Antibodies**	*Teplizumab*	2	2 years	At 2 years, patients in the treatment group had less reduction in MMTT-stimulated C-peptide AUC when compared to the control group (17).
		3	2.5 years	At a median follow up of 2.5 years, 50% of the teplizumab-treated population remained in stage 2 T1D compared to 22% of the placebo group (21).
**Anti-CD20 Monoclonal Antibodies**	*Rituximab*	3	1 year	At 1 year, the mean MMTT-stimulated C-peptide AUC was significantly higher in the rituximab group than in the placebo group (23).
**Anti-IL-21 & GLP-1 agonists**	*Anti-IL-21 & Liraglutide*	3	54 weeks	At 54 weeks, those receiving Anti-IL-21 and liraglutide had 48% higher MMTT-stimulated C-peptide levels when compared to placebo, representing only a 10% decrease from baseline (72).
**Anti IL-12 & IL-23 Monoclonal Antibody**	*Ustekinumab*	3	1 year	At 1 year, those receiving the intervention had 49% higher MMTT-stimulated C-peptide levels (27)
**Dimeric Fusion Protein**	*Alefacept*	3	2 years	At 2 years, the alefacept group had lower insulin requirements, fewer hypoglycemic episodes and higher MMTT-stimulated C-peptide levels when compared to the control group (30)
**Anti-Thymocyte Globulins (ATG)**	*Thymoglobulin*	3	2 years	A 2-year MMTT-stimulated C-peptide AUC was significantly elevated in ATG versus placebo, but not in ATG+GCSF versus placebo. Both ATG and ATG+GCSF were associated with reduced HbA1c at 2 years (32).
**Calcium Channel Blockers**	*Verapamil*	3	1 year	The treatment group had a 30% higher MMTT-stimulated C-peptide AUC (45).
**CTLA-4 Analogs**	*Abatacept*	3	2 years	At the 2 year follow up, MMTT-stimulated C-peptide AUC was found to be 59% higher in the treatment vs placebo group (40).
**JAK Inhibitors**	*Baricitinib*	3	48 weeks	Daily treatment over 48 weeks was associated with an increased meal-stimulated mean C-peptide level (36).
**Tumor Necrosis Factor Alpha (TNF-α) Blockers**	*Golimumab*	3	1 year	At 1 year, the MMTT-stimulated C-peptide AUC remained higher in the treatment versus placebo group (37). C-peptide AUC decreased 12% with golimumab compared to 56% with placebo.
**Tyrosine Kinase Inhibitors**	*Imatinib*	3	2 years	The treatment group had a higher MMTT-stimulated C-peptide AUC at 1 year, but this effect was not sustained at 2 years (41).
**Neurotransmitter and antigen-based therapy**	*GABA and GAD-alum*	3	1 year	No change in glycemia, fasting or meal-stimulated C-peptide AUC at 1 year. Mean fasting glucagon levels did not increase in the GABA or GABA/GAD-alum groups and meal-stimulated glucagon levels were lower in the intervention groups (50)
**Autologous Dendritic Cell Therapy**	*AVT001*	3	1 year	At 1 year, there were no differences in HbA1c or insulin dose, but there was less decline in C-peptide production (52)
**Autologous Mesenchymal Stem Cells (MSC)**	*Autologous bone marrow derived MSCs*	3	1 year	Those receiving MSCs had reductions in level 1 and level 2 hypoglycemia, and fewer hypoglycemia events. Earlier treatment (within the first year) was associated with lower HbA1c levels at 1 year when compared to later treatment (at least 1 year after T1D diagnosis) (53)

*AUC, Area Under the Curve; MMTT, mixed meal tolerance test; HbA1c, hemoglobin A1c; GCSF, granulocyte colony-stimulating factor.

The authors apologize for these errors and state that this does not change the scientific conclusions of the article in any way. The original article has been updated.

